# Carbon Fiber Prepreg Composites Failure Mechanism Based on Electrical Resistance Method during Hight-Strain Rate Loading

**DOI:** 10.3390/polym15030484

**Published:** 2023-01-17

**Authors:** Hongji Zhang, Yang Han, Yuanyuan Ge, Zhiyong Sun

**Affiliations:** 1 School of Energy Engineering, Yulin University, Yulin 719000, China; 2 School of Mechanical Engineering, Shaanxi University of Technology, Hanzhong 723001, China

**Keywords:** carbon prepreg, filament winding, Hopkinson impact, pressure resistance, composite materials

## Abstract

In this study, a unidirectional and plain weave carbon fiber/epoxy prepreg was used as the raw material, and the prepreg tape winding process was used to prepare carbon fiber/epoxy prepreg composites with 65% and 75% carbon fiber volume content, respectively. Based on traditional damage experiments and mechanical measurements, electrical measurements are introduced to study the damage to carbon fiber prepreg composites. The damage behavior of the carbon fiber prepreg composite under a high-speed impact load was monitored using the resistance method. By arranging electrodes on the sample and tracking the change in resistance during the entire process of high-speed impact of the material, the relationship between the damage and the change in resistance parameters of the carbon fiber prepreg composite winding products under high-speed impact was determined. The stress-strain curve and the final failure mode of the sample and the microstructure mechanics of carbon fiber prepreg winding products under different strain rates were analyzed. These results indicate that, as the change in resistance over time was almost stable from 0 to 200 μs. From 200 to 250 μs, the resistance decreases sharply; from 250 to 400 μs, the resistance approximates a plateau. From 400 to 500 μs, the resistance value increases again; at this time, the resistance value decreases to 3.2% of the initial resistance value.

## 1. Introduction

Carbon fiber/epoxy matrix composite comprises of carbon fiber/epoxy prepreg as the raw materials and it is laminated at a certain angle under the effect of process parameters (tension, pressure, temperature, and linear velocity). It has a high specific modulus, specific strength, and creep resistance. It has been widely used in the aerospace, energy, chemical, and automobile industry industries [1,2]. The carbon fiber/epoxy matrix composite is sensitive to impact. When impacted by foreign objects, the surface or interior of the material are easily damaged. In particular, matrix cracking or delamination damage occurs in the interior of the material. However, the surface hardly exhibits any damage defects. These internal damages and failures significantly degrade the mechanical properties of materials, and the strength decreases by approximately 35–40%. Consequently, the bearing capacity of the structure is significantly reduced, which may pose a potential threat to the overall damage and failure of the structure. Generally, carbon fiber/epoxy matrix composites are composed of conductive carbon fibers (resistivity ρ = (1.0–1.5) × 10^−3^ Ω·cm) and insulating epoxy resin (resistivity ρ = 1.0 × (10^15^–10^17^) Ω·cm) [3,4]. Since the volume content of carbon fiber in the prepreg composite is more than 50% in actual cases, carbon fiber is distributed in the matrix as a bundle or interlaced warp and weft to form a conductive network. Thus, the carbon fiber/epoxy matrix composite also exhibits conductivity. The conductive mechanism of the carbon fiber/epoxy matrix composite is through the joint action of contact and seepage between carbon fibers, and any change in the contact between carbon fibers directly affects the apparent resistance. When the carbon fiber/epoxy matrix composite products are damaged by external forces, it results in damage, such as material delamination and fiber fracture, which inevitably causes a change in the material resistance [5,6].

Multidirectional laminated carbon fiber composite is a type of unidirectional carbon fiber laminated in a specific direction. According to the number and direction of the layers, the resistance of the multi-directional laminated carbon fiber composite can be regarded as quasi-isotropic. Hart [7] used the 4-probe electrical resistance of carbon-fiber-reinforced polymer (CFRP) composites as a metric for sensing low-velocity impact damage. A robust method was developed to recover directionally dependent electrical resistivities using an experimental line-type 4-probe resistance method. Wen [8] reviewed the recent advances in damage detection in CFRPs using resistance measurements. In addition to the discussions on various experimental methods for self-sensing and damage detection, modeling from microscale to continuum levels for the prediction of resistance changes due to mechanical damage is also presented. Future directions for damage detection of CFRPs using the resistance method are provided. Xia [9] established an analytical model for the transverse (perpendicular to the fibers) electrical resistance of pristine and damaged unidirectional composites, complementing previous reports on longitudinal resistance. This study demonstrates that both longitudinal and transverse resistance changes are sensitive to damage in a predictable manner and can be used together to improve the reliability of damage assessment during the loading of CFRPs. Todoroki [10] discussed the use of electrical resistance and potential change in various methods for monitoring CFRP composites. The discrepancy is discussed in detail in this study. To monitor damage, such as delamination cracks, the electrical resistance change method is used to accurately estimate the location and dimension of the damage. This study also discusses electrical potential change and eddy current methods for monitoring CFRP composites. Mirjavadi S. [11,12,13,14] investigated the truncated conical shell segments made from multi-scale epoxy/carbon nanotube/fiberglass material are studied in this research in the view of evaluating nonlinear free vibration behavior. Transient vibration responses of a porosity-dependent functionally graded nanobeam under different impulsive loadings have been investigated in the context of non-local strain gradient theory. Three impulse loads of rectangular-type, linear-type and sine-type have been applied to top surface of nanobeam.

Abry [15] investigated the possibility of in situ detection of damage in unidirectional CFRP using electric resistance measurements, and the conducting paths in unloaded samples were investigated by changing the electrode location. Quaresimin M. [16] an analytical model to predict the laminate stiffness as a function of the damage scenario is derived and later combined with the electric one, thus establishing a direct correlation between the resistance change and the stiffness degradation. Suo T. and Li T. [17] studied the mechanical behavior of two-dimensional (2D) carbon fiber reinforced silicon carbide composites, and conducted experimental studies on quasi-static and dynamic uniaxial compression in the temperature range of 293–1273 K. The experimental results indicated that the strain rate has a significant effect on the compressive properties of 2D C/SiC composites. Li Y. l. [18] used the modified Hopkinson pressure method to study the compression behavior at 500/s strain rate. The results indicated that the strength and stiffness improved at high strain rates. 

Quaresimin M. [18] in this chapter, a framework developed by the authors to predict the damage evolution and the stiffness loss in multidirectional laminates under cyclic loadings is presented. It integrates models and criteria for predicting crack initiation and propagation under multiaxial fatigue loadings, the stress re-distributions and the stiffness loss in the presence of off-axis cracks, and a procedure to predict the crack density evolution. Quaresimin M. [19] the novel all-composite sandwich panel with channel core was proposed for simultaneous compressive strength and energy absorption characteristic subjected to quasi-static out-of-plane compression. The structure was composed of carbon fiber reinforced polymer (CFRP) face-sheets and channel core. The compressive responses and deformation mechanism of the proposed structure were studied by the method of experiment and finite element analyses (FEA). 

In this study, based on the self-conductivity of a carbon fiber/epoxy matrix prepreg composite, the generation and expansion of damage during high-speed impact were monitored online using the electrical resistance change method. The resistance of the carbon fiber/epoxy matrix prepreg composite varied with the development of damage at high strain rates. By arranging electrodes on the sample and tracking the change in resistance during the entire process of the high-speed impact of the material. In addition, the high-speed impact test process of the Hopkinson compression bar was deeply and systematically analyzed, and the relationship between the resistance change and the damage of the carbon fiber/epoxy matrix prepreg composite was clarified using the change in resistance to monitor the safe use of the carbon/fiber epoxy matrix prepreg composite.

## 2. High-Speed Impact Test

### 2.1. Principles and Requirements of High-Speed Impact Test

**Figure 1:**
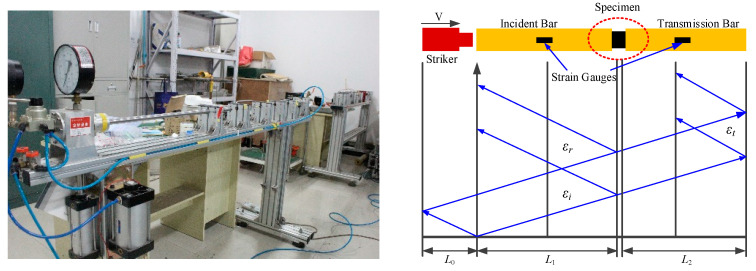
Schematic diagram of split Hopkinson compression bar device and stress wave transmission.

Figure 1 shows the Hopkinson pressure bar experimental system, which is mainly composed of the impact, incident, and transmission bars, and is also equipped with an air pressure driving device and data acquisition equipment. Generally, the impact, incident, and transmission bars are made of high-strength metallic materials of the same material and have the same diameter. Before the experiment, the impact, incident, and transmission bars were placed horizontally and maintained in the same axis. The sample was clamped between the incident bar and transmission bar and maintained on the same axis as the incident bar and transmission bar. In the experiment, the striker was driven by high-pressure gas and it impacted the incident bar at a certain speed, and the generated stress wave propagated along the incident bar and was recorded by the strain gauge on the incident bar (recorded as incident wave). When the wave continued to propagate to the contact surface between the incident bar and the sample, the sample was loaded.

The SHPB experimental system is based on one-dimensional (1D) stress wave theory, which indirectly obtains the load and displacement histories of the two ends of the sample during deformation. The principle of SHPB test system is shown in Figure 1, detailed experimental principles in literature [20]. According to the one-dimensional stress wave propagation theory in uniform slender bar and the assumption of the uniformity of stress and strain in the sample, the calculation equations for the average stress, average strain and average strain rate of the sample under impact loading can be obtained.

(1) $$\sigma_{s}=\frac{E_{b}A_{b}}{A_{s}}\varepsilon_{t}$$

(2) $$\dot{\varepsilon }=-\frac{2c_{0}}{l_{s}}(\varepsilon_{i}-\varepsilon_{t})$$

(3) $$\varepsilon =-\frac{2c_{0}}{l_{s}}\int_{0}^{t}\left(\varepsilon_{i}-\varepsilon_{t}\right)dt$$

where *ε_i_* and *ε_t_* are incident wave and transmitted wave strain, respectively. *σ*_s_, *ε*, $\dot{\varepsilon }$ are the stress, strain and strain rate of the sample, respectively. *l_s_*, *A_s_*, and *A_b_* are the sample length, sample sectional area and rod sectional area, respectively. $c_{0}=\sqrt{E_{b}/\rho_{b}}$, $E_{b}$, $\rho_{b}$ are the elastic wave velocity, elastic modulus and density of the rod, respectively.

### 2.2. Experimental Materials and Sample Preparation

The prepreg material used in the experiment was provided by the Weihai Guangwei Composite Materials Co., Ltd(Weihai, China). This type of prepreg uses T700-12K carbon fiber (Toray Japan) as the reinforcing material, and the epoxy matrix was prepared by uniformly mixing JF-43 epoxy resin(Wuxi, China). The test sample was made of a prepreg tape winding process and then cured by autoclaving, according to the characteristics of epoxy resin materials, the curing process is as follows: heat preservation for 1 h at 90 °C and 110 °C, respectively, and for 4 h at 130 °C. In addition, the size of the sample was 16 mm (outer diameter) × 13 mm (inner diameter) × 16 mm (height). The relationship between the high strain rate impact damage of the carbon fiber/epoxy matrix composites and the change in the resistance signal obtained using a SHPB is shown in Figure 1. The Hopkinson device includes a striker (that is, an impact bar (400 mm)), an incident bar (2000 mm), and a transmission bar (2000 mm), each with a diameter of 19 mm. Before testing, the sample was sandwiched smoothly between the incident bar and the transmission bar, and it was strictly ensured that the incident bar, transmission bar, and the sample were on the same line (meeting 1D wave propagation theory), and the mechanical response of the specimen under a high strain rate was mainly reflected by the change in the transmitted wave waveform. 

**Figure 2:**
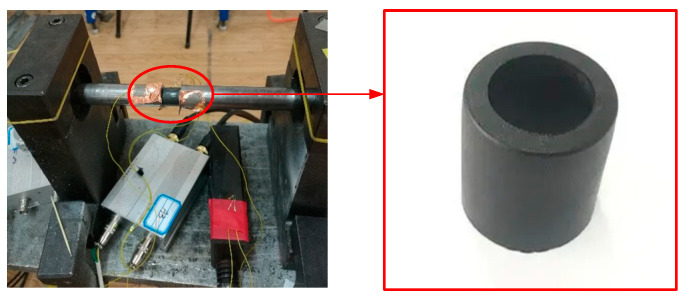
Experimental samples and installation diagram.

From Equations (1)–(3), according to the strain signals collected by the strain gauges attached to the incident bar and the transmission bar, the stress-time, strain-time, and strain-rate-time curves of the material at different high strain rates can be obtained, and finally the stress-strain curves are obtained. To study the strain rate sensitivity of the mechanical response of carbon fiber/epoxy matrix composites, three different air pressures (0.3, 0.5, and 0.7 MPa) were used to drive the striker, and the incident bar was impacted at different speeds. The experimental samples and installation methods are shown in Figure 2. The square incident wave is generated after the striker hits the incident bar, and the transmitted wave signal transmitted through the sample is recorded by a resistance strain gauge. After the time-displacement process, the strain on both sides was obtained, the resistance signal was subsequently converted into a voltage signal by an ultra-dynamic strain gauge and amplified, and the data and voltage-time curves were recorded using a PC12406 data acquisition device. 

### 2.3. Measurement of Resistance Signal during High-Speed Impact

**Figure 3:**
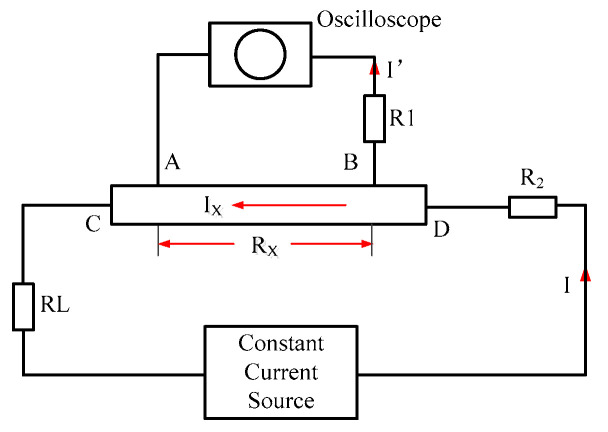
Schematic diagram of the four-electrode lead measurement of resistivity.

During the experiment, the four-electrode lead method was used to measure the resistance signal. The test circuit is shown in Figure 3. The Rx denotes the resistance of the sample to be measured; A and B are the output endpoints of the measured voltage signal; C and D are the current input terminals; R_1_ and R_2_ are the equivalent resistances of the contact resistance at the output and input; R_L_ is the cable impedance; and I_x_ and I’ are the currents flowing through the sample and the oscilloscope, respectively. The contact resistance R_2_ of the sample and the remaining resistances other than the A and B terminals of the sample can be considered as the cable resistance R_L_, and their influence is eliminated by the constant-current source. For the effect of the lead at the output and the contact resistance R_1_, because the input impedance of the oscilloscope is significantly larger than the resistance to be measured R_x_, the shunt I’ is very small, and the voltage division on R_1_ is also very small. The signal on the oscilloscope was approximately equal to the true voltage drop across the sample, thus ensuring high measurement accuracy.

Given the sample configuration and 1D strain compression properties of a plane impact wave, it can be approximated that the length and width of the sample remain unchanged under the compression of the impact wave, and the resistivity ρ_F_ of the sample is determined as follows:

(4) $$\rho_{F}=\frac{R_{F}\mathrm{XZ}}{L}(\frac{V_{F}}{V_{0}})$$

where R_F_ denotes the resistance value of the sample obtained through experimental measurement, L is the distance between the two electrodes of the output voltage signal, X and Z are the initial width and thickness of the sample, respectively, and V_F_/V_0_ is the compression ratio of the sample when the impact wave pressure is balanced. In the actual calculation process, because the sample is measured under the condition of a pulsed constant current source, R_F_/R_0_ = ∆V_F_/∆V_0_

(5) $$\rho_{F}=\frac{\Delta V_{0}}{\Delta V_{F}}(\frac{V_{F}}{V_{0}})\rho_{0}$$

where ΔV_F_ and ΔV_0_ denote the final state voltage drop and the initial voltage drop across the sample, respectively.

## 3. Experimental Results and Analysis

### 3.1. Variation of Resistivity with the Impact Process

**Figure 4:**
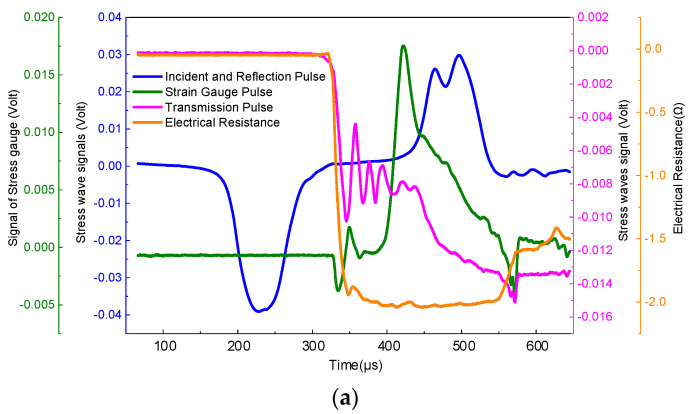

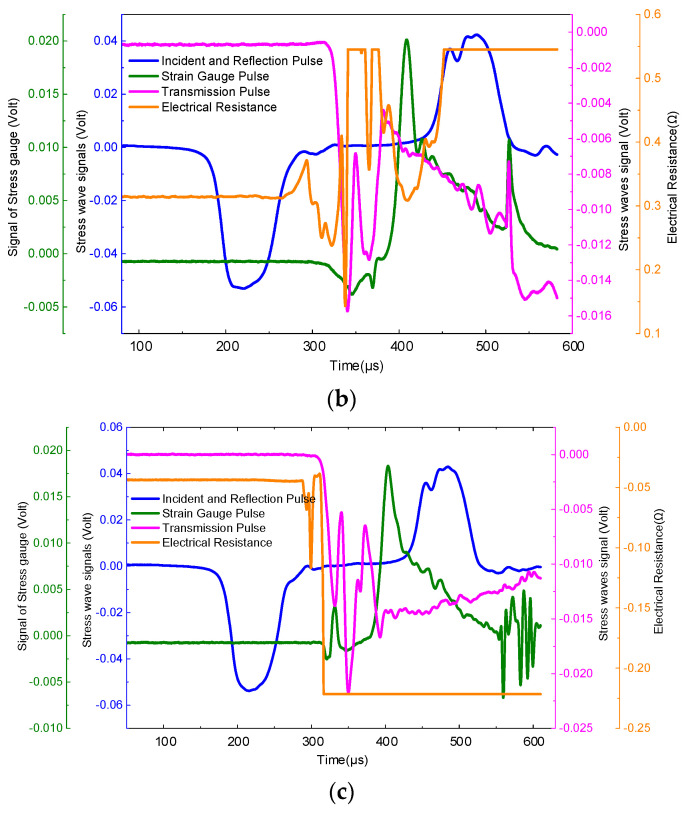
Hopkinson impact test results of unidirectional prepreg composites. (**a**) The driving pressure of the striker is 0.3 MPa. (**b**) The driving pressure of the striker is 0.5 MPa. (**c**) The driving pressure of the striker is 0.7 MPa.

**Figure 5:**
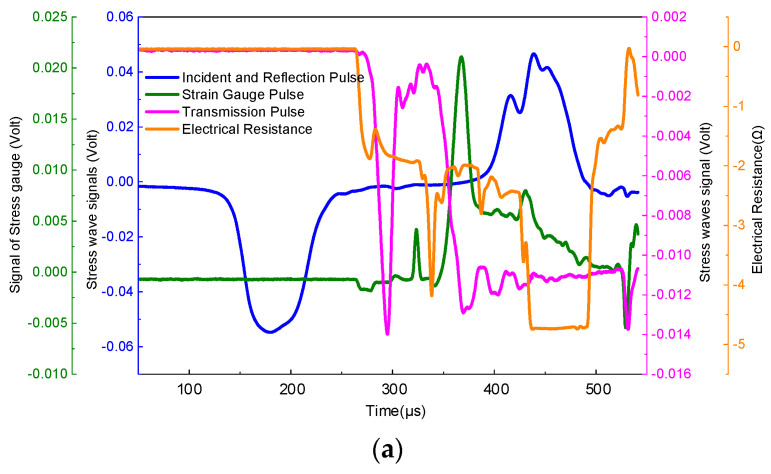

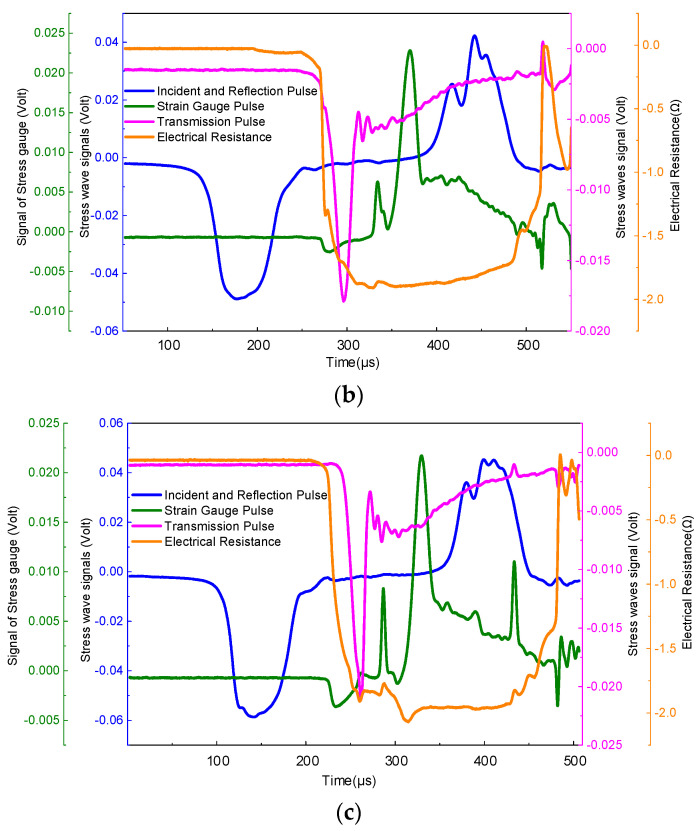
Hopkinson impact test results of plain weave prepreg composite. (**a**) The driving pressure of the striker is 0.3 MPa. (**b**) The driving pressure of the striker is 0.5 MPa. (**c**) The driving pressure of the striker is 0.7 MPa.

During the Hopkinson pressure bar experiment, the driving air pressure of the striker was set at 0.3, 0.5, and 0.7 MPa, and variations of longitudinal impact damage and electrical resistance of plain weave and unidirectional prepreg tape wound products under different air pressure conditions were tested. In addition the results are shown in Figure 4 and Figure 5. The impact wave entered the sample at time zero, and the sample passed out after approximately 3.3 μs. Figure 4 and Figure 5 show that when the striker hits the incident bar, the resistance of the sample does not change. The voltage signal of the slowly increasing voltage appeared on the oscilloscope only after the impact wave passed out of the sample interface for approximately 200 μs. Since the resistance method is a very sensitive measurement method, the response time for measuring the resistivity of the sample under impact-wave compression is only in the order of microseconds. Therefore, once the resistivity of the sample changes under the compression of the impact wave, the voltage test signal suddenly increases at the surface after the impact wave passes out of the sample. Figure 4a shows that when the driving pressure of the striker is 0.3 MPa, the resistance signal is almost stable in the early stage of the impact. With the transmission of the transmitted wave signal, the resistance signal of the material suddenly began to decrease sharply. At the early stage of fiber fracture, it reached a minimum, and a sudden increase occurred when the fiber broke completely. Subsequently, it reached a steady state for a certain period of time. With the increase in the reflected wave signal, the material resistance signal also exhibited a gradually increasing trend and finally fluctuated within a certain amplitude range. When the driving pressure of the strike is 0.5 MPa, the trend of resistance decreases before the damage can be detected, and a series of spikes appear during the late oscillation of the resistance signal, indicating that at this time, the internal microstructure of the material changes before destruction, as shown in Figure 4b. Comparison of Figure 4 and Figure 5 demonstrate that the resistance variation law of plain weave and unidirectional prepreg composites is different under high-strain conditions. Figure 5a–c show that for plain weave prepreg composites, when the driving pressure of the strike is less than 0.5 MPa, the longitudinal volume resistance increases with the increase in the driving air pressure of the impact rod. This is because the elastic elongation of the carbon fiber was very small, and the change in the sample volume resistance due to the size effect was very small. When the driving pressure of the strike reaches 0.7 MPa, the longitudinal volume resistance decreases sharply, mainly because of the increase in the density of carbon fibers arranged in the 0° direction in the unidirectional prepreg composites.

Figure 4 and Figure 5 show that the change trend of the stress of the sample with time is the same regardless of the impact speed of the striker. Since the relationship between stress and time reflects the change in the overall load on the sample, and the change in resistance reflects the structural changes of the carbon fiber inside the sample during the impact process, with an increase in the emission pressure of the striker, the change in resistance can keep up with the changes in the carbon fiber structure. Thus, the sensitivity of the resistance to changes in the compression process of the epoxy matrix increases. 

### 3.2. Analysis of the Relationship between Resistance Changes and Structural Changes during Hopkinson Impact

**Figure 6:**
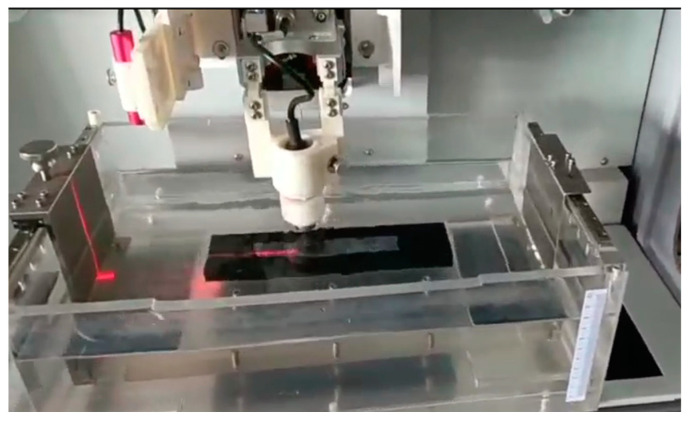
Water immersion ultrasonic testing system.

**Figure 7:**
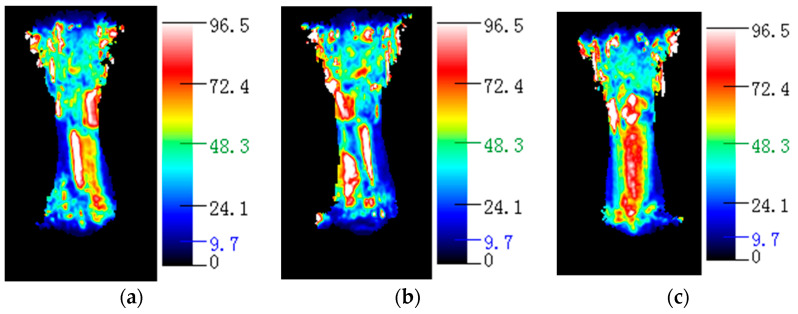
Ultrasonic test results of unidirectional prepreg composites in different emission pressure of the striker. (**a**) 0.3 MPa. (**b**) 0.5 MPa. (**c**) 0.7 MPa.

**Figure 8:**
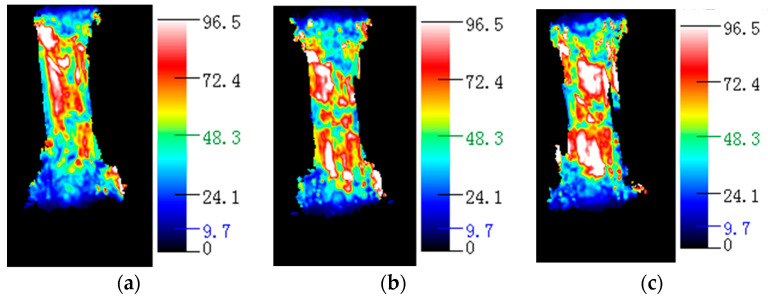
Ultrasonic test results of plain weave prepreg composite in different emission pressure of the striker. (**a**) 0.3 MPa. (**b**) 0.5 MPa. (**c**) 0.7 MPa.

**Figure 9:**
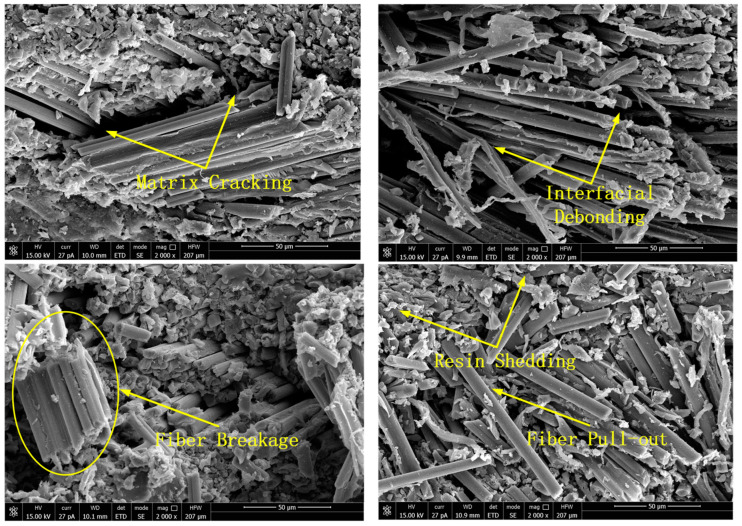
SEM image of the inner layer of the impact sample.

The failure mechanism of the carbon fiber/epoxy matrix composite material when the emission pressure of the striker is 0.3 MPa is investigated in detail. Figure 4 and Figure 5 show that the change in resistance over time was almost stable from 0 to 200 μs. From 200 to 250 μs, the resistance decreases sharply. From 250 to 400 μs, the resistance approximates a plateau. From 400 to 500 μs, the resistance value increases again. At this time, the resistance value decreases to 3.2% of the initial resistance value. At 400 μs, the material was damaged, and the resistance increased sharply to a maximum, which was less than the initial resistance. From this change in the resistance and stress-time curve, it is inferred that the internal microstructure of the material changes as follows: In the time period from 0 to 200 μs, it is mainly the joint elastic compression of the matrix and the fiber. However, owing to the small amount of compression, the resistance change owing to the size effect is not significant, and the resistance is basically stable. In the period from 200 to 250 μs, owing to the large difference in the elastic modulus of the epoxy matrix and carbon fiber, compression failure of the matrix and shear failure of the fiber-matrix interface occurred in a short time. In the process of reducing the resistance, if the impact speed is very low, a series of peak fluctuations occur in the resistance, as shown in Figure 4 and Figure 5. This fluctuation reflects the gradual fracture of carbon fibers. At 400 μs, the material was damaged, the conductive network was destroyed, and resistance suddenly increased. A water immersion ultrasonic testing system (UPK-T48) was used to detect the size and location of the damage of the unidirectional and braided prepreg composite materials after impact at different emission pressure levels of the striker, as shown in Figure 6, Figure 7 and Figure 8. The matrix was deboned by carbon fiber. This phenomenon can be observed in the electron microscope photographs of the inner layer of the sample. Figure 9 shows that there is significant matrix chipping and interface debonding. The carbon fiber originally embedded in the resin had a certain space for movement. Some off-axis carbon fibers were aligned, the alignment of carbon fibers in the 0° direction increased, and the contact tightness between some carbon fibers increased, which reduced the volume resistance in the longitudinal direction and sharply decreased the resistance. In the time period from 250 to 400 μs, the increase in the alignment of carbon fibers in the 0° direction increases the volume resistance in the longitudinal direction, and the compression of carbon fibers reduced the volume resistance in the longitudinal direction. The combination of the two slows the resistance decrease trend, and the resistance appears on the platform. In the period from 400 to 500 μs, most of the matrix was broken, and the carbon fiber bore most of the load. Since the compression rate of the carbon fiber is very low, continued compression causes most of the carbon fiber to break. The parallel-aligned carbon fibers were close to each other and overlapped. Figure 9 shows that the conductive path changed from multiple parallel paths to a network, resulting in a sharp reduction in the volume resistance in the longitudinal direction. 

### 3.3. Correspondence between Stress Wave Propagation and Resistivity

**Figure 10:**
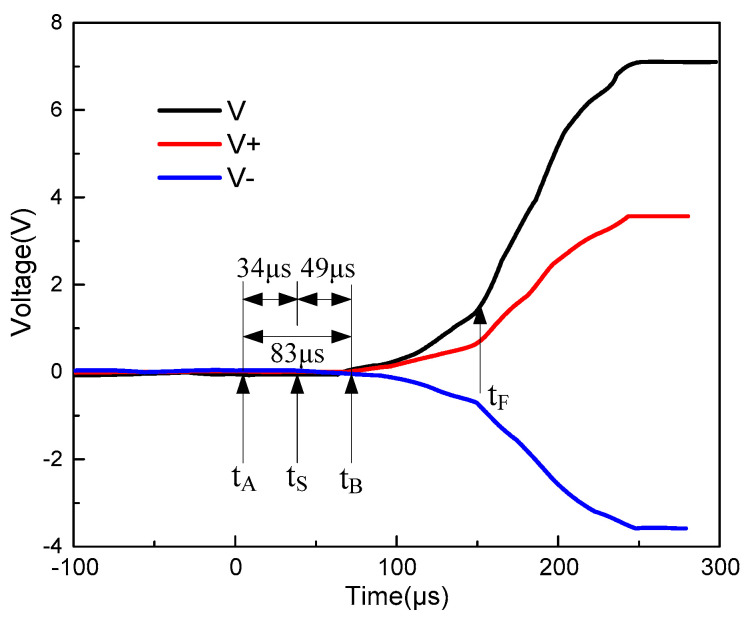
Three characteristic time domains of voltage signal changes.

In order to observe the conductive behavior of carbon fiber/epoxy matrix composite materials under the action of impact waves in more detail, the correspondence between the failure wave propagation and resistivity was analyzed. Figure 10 shows the signal measured under the condition that the pressure of the striker is 0.3 MPa. The t_A_, t_S_, t_B_, and t_F_ denote the moments when the stress wave enters the sample, reaches the incident bar electrode, conduction between the electrodes begins, and the stress wave reaches the transmission bar electrode, respectively.

The figure shows that the voltage signal starts to increase at time t_B_. That is, the impact wave begins to occur between the two electrodes approximately 83 μs after entering the sample. In the design of the experimental sample, the distance between the incident bar electrode and collision surface was 2 mm, and the impact wave velocity was 4853 m/s. That is, the time required for the impact wave to propagate into the bar electrode was 34 μs, and there was a time difference of approximately 49 μs between it and 83 μs. According to previous measurement results, the microcrack propagation speed between the two electrode holes was approximately 2.98–5.37 km/s, and the distance between the two electrodes was 20 mm. Therefore, the time between the dynamic crack propagation between the two electrodes until they intersect is approximately 38–67 μs. Based on this analysis, the change in the voltage signal shown in Figure 10 can be divided into three time periods. (1) From time t_A_, when the incident bar hits the sample, to time t_B_, the voltage signal is zero, and there is no conduction between the two electrodes. (2) Between t_B_ and t_F_, the voltage signal started to increase, indicating that the dynamic crack growth between the two electrodes began to cause electrical conduction. (3) When the destruction wave propagates to the electrode of the transmission rod, it causes a change in the measured signal, an inflection point appeared in the voltage signal after t_F_. Figure 10 shows that the voltage signal exhibits a relatively clear inflection point. The slope of the straight line illustrated in Figure 10 indicates that when the destruction wave reached the end of the transmission bar electrode, the conductivity between the two electrodes increased. The experimental results illustrate the effect of stress wave propagation on the resistivity decrease in carbon/fiber epoxy matrix composites and further show that the physical mechanism of resistivity decrease is the propagation of micro-cracks.

### 3.4. Stress-Strain Curve Analysis

**Figure 11:**
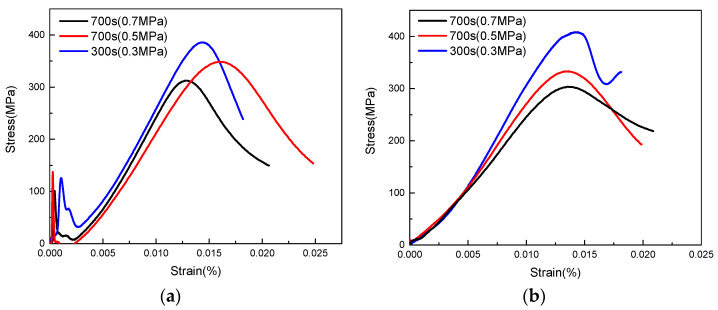
Stress-strain curves of two materials under high-speed impact. (**a**) Unidirectional prepreg composites. (**b**) Plain weave prepreg composite.

A typical stress-strain curve of the high-speed impact of the carbon fiber/epoxy matrix composites is shown in Figure 11. Figure 11 shows that at the initial stage of the stress-strain curve, the relationship between the stress and strain exhibits a positive correlation. Under dynamic loading conditions, the stress-strain relationship of carbon fiber/epoxy matrix composites is nonlinear. As the strain rate increased, the failure strength increased, failure strain decreased, and elastic modulus increased. Figure 11 shows that the strengths of the unidirectional carbon fiber prepreg composite under different strain rate conditions were 303.27 MPa, 332.78 MPa, and 407.73 MPa. In contrast, the strength of plain weave carbon fiber prepreg composite materials was 331.99 MPa, 348.52 MPa, 385.69 MPa. These results indicate that as the strain rate increased, the strength of both materials increased. In comparison with the quasi-static impact compression, the minimum strengths of the two materials increased by 43.7% and 46.15%, respectively. This shows that the fiber arrangement has a significant influence on the mechanical properties of the material under longitudinal compression. This is mainly due to the weaving angle of the woven prepreg when the fiber is compressed in the longitudinal direction, and the fiber bundle has a certain angle with the compression direction. Carbon fiber/epoxy matrix composites exhibit different strength characteristics under static and dynamic compression. Since the matrix is a brittle material, its dynamic damage variables are affected by strain and strain rate. That is, under high-strain-rate compression, the impact sensitivity of the brittle material causes the matrix to damage first. The conclusion is consistent with in literature [21]. With an increase in strain and accumulation of matrix damage, the stress growth reduces, indicating non-linear mechanical behavior characteristics.

**Figure 12:**
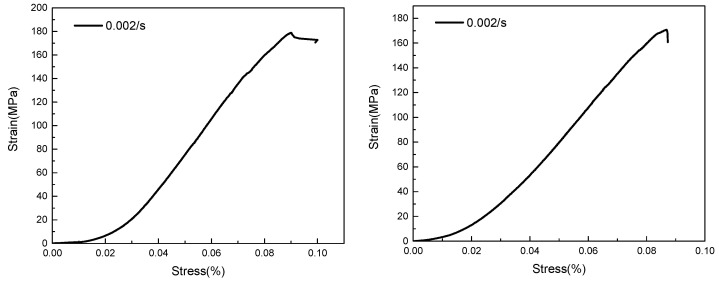
Stress-strain curves of two materials under quasi-static compression.

Figure 12 shows the compressive stress-strain curves of the carbon fiber/epoxy matrix composites under quasi-static conditions. The failure strain of the material under static compression was 0.0899 and 0.08685, and the corresponding ultimate strengths were 170.76 MPa and 178.85 MPa, respectively, indicating that the materials exhibit brittle characteristics. This is because of the initial micro-defects contained in the material, and the material damage during the loading process slowly accumulates and develops. Under dynamic compression conditions, the elastic section becomes shorter and the strength of the material increases. 

## 4. Conclusions

The changes in the resistance of unidirectional and woven prepreg wound products under Hopkinson impact conditions were compared, and the damage behavior of the materials during impact was verified. The results indicated that during the entire failure process, if the Hopkinson shock pressure was low, a series of peaks in the electrical resistance appeared, which was caused by the gradual fracture of some weak carbon fibers. Finally, the sample was compressed and destroyed, the conductive network was completely destroyed, and the resistance suddenly increased. At the end of the impact process, the increase in resistance was caused by the fracture of the carbon fiber.

From the three characteristic time domains of the voltage signal change during the Hopkinson shock process, it was observed that the stress wave only began to occur between the two electrodes approximately 83 μs after entering the sample. The time required for the dynamic crack propagation between the two electrodes to intersect with each other was approximately 38–67 μs. The essence of stress waves in CF/epoxy matrix composites is that the sample loading surface micro-cracks propagate into the sample under the effect of stress waves. When the damage wave propagates to the electrode of the transmission bar, it causes a change in the measurement signal, further indicating the resistivity of the material. 

According to the stress-strain curve of the material after impact, it was observed that the material strain rate effect was significant, and the material exhibited more clear brittle failure characteristics under dynamic loading. In comparison with quasi-static compression, the damage strength was increased, failure strain was reduced, elastic modulus was increased during dynamic impact, and elastic modulus had a linear relationship with the strain rate. As the strain rate increased, the strength of both materials increased. In comparison with the quasi-static impact compression, the minimum strengths of the two materials increased by 43.7% and 46.15%, respectively.

## Data Availability

The data presented in this study are available on request from the corresponding author.
